# Can bacterium UD1023 lessen the uptake and bioaccumulation of heavy metals in plants? An update

**DOI:** 10.17179/excli2015-661

**Published:** 2016-01-06

**Authors:** Kamal Niaz, Faheem Maqbool, Haji Bahadar, Mohammad Abdollahi

**Affiliations:** 1 Department of Toxicology and Pharmacology, Faculty of Pharmacy and Pharmaceutical Sciences Research Center, Tehran University of Medical Sciences, International Campus, Tehran, Iran; 2 Department of Pharmacy, Kohat University of Science and Technology Kohat, Pakistan

## Abbreviations

Ni - Nickel; Cd - Cadmium; TL - Thallium; As - Arsenic; DEHP - Di-(2-ethylhexyl) phthalates; DBP - Di-n-butyl phthalates; AMF - Arbuscular mycorrhizal fungi; MHB - Mycorrhizal helping bacteria; PGPR - Plant growth promoting rhizobacteria; ↑ - Increased; ↓ - Reduced 

## ⁯

Pollution of water and soil with heavy metals highlights one of the most important public health threats. Soil and plants are polluted with heavy metals like nickel (Ni), cadmium (Cd), thallium (Tl) and arsenic (As) which mostly comes from the irrigation system, chemical industry, agrochemicals, and pesticides in the environment. The plant root signifies the first barrier to the selective accumulation of ions and heavy metals present in the soil. Kinetic data, uptake for nutrient ions and chemically related nonnutrient analogs suggest that metabolic processes associated with root absorption of nutrients regulate both rate of absorption and the affinity of specific nonnutrient ions. Differnet detailed kinetic studies of Ni, Cd, and Tl uptake by intact plants demonstrate multiphasic root absorption processes over a wide range of concentration (Cataldo and Wildung, 1978[4]). For example, wheat and some vegetables have been reported to bio-accumulate heavy metals more than WHO/FAO permissible level. In addition, excessive application of pesticides and herbicides in the agriculture for the protection of plants from diseases and high production is also a threat to humans (Bahadar et al., 2014[2]). Some of the trace elements (phosphorus, nitrogen, potassium) are necessary for the plant growth, but with that plants also take noxious metals and metalloids. The metals concentrations are different among various plants species and body parts. A study conducted in the Hamadan Province, Iran proves that metals (copper, zinc, iron and magnesium) accumulation depends on different factors like metals concentration, pH, electrical conductivity, nutrients in the subsoil (substrata). The results showed that zinc and copper concentration in aboveground and underground tissues plants were significantly positive related to their total subsoil amount and soil phosphorus had negative affectson copper, iron and zinc (Nouri et al., 2009[12]). The bioaccumulation of the phthalates and their metabolites like Di-(2-ethylhexyl) phthalates (DEHP) and Di-n- butyl phthalates (DBP) in some medicinal plants and agricultural crops (radish, wheat) which is a human carcinogen, having adverse effects on different organs, reproductive and developmental anomalies (Saeidnia and Abdollahi, 2013[13]).

## The Role of Microbes

Microbes present in the rhizoshperes region of soil play an important role in the phytoremediation of the metals (Khan, 2005[8]). Mostly, in the nonagricultural surroundings, arbuscular mycorrhizal fungi (AMF), mycorrhizal helping bacteria (MHB), p-solubilizing bacteria, and plant growth promoting rhizobacteria (PGPR) are helping to sustain the soil fertility than in conventional agriculture and forestry, where constant and high use of pesticide and herbicide minimize their significance. These microbes twitch their work when significant concentration is achieved like quorum sensing. AMF produce high amount of insoluble glomalin and glycoprotein which impound trace elements and play role in the maintenance of contaminated soils (Khan, 2005[8]). Furthermore, the role of some important soil microbes to reduce or immobilize the metals, their transclocation in the plants is shown in the Table 1(Tab. 1) (References in Table 1: Tripathi et al., 2005[18]; Tank and Saraf, 2009[17]; Li et al., 2010[9]; Joshi and Juwarkar, 2009[7]; Gonzalez-Chavez et al., 2004[6]; Yang et al., 2012[19]; Aafi et al., 2012[1]). This means that phytoremediation is a low cost strategy, tolerance to disease in situ technology applied to remove or control heavy metals in the soils. 

However, high concentration of heavy metals adversely effects the plant growth. As there are different role of heavy metals in the plant including their role as electron carrier, catalyzing enzymatic activities, in the redox reactions, and their presence in the structure of DNA and RNA. In addition, heavy metals also influence the functionality of enzymes as well as protein. The functionality, activity and permeability of plasma membrane of the plants are effected by heavy metals. The oxidative stress of heavy metals also influences the plants growth by production of reactive oxygen species (Miransari, 2015[11]).

**Figure 1:**
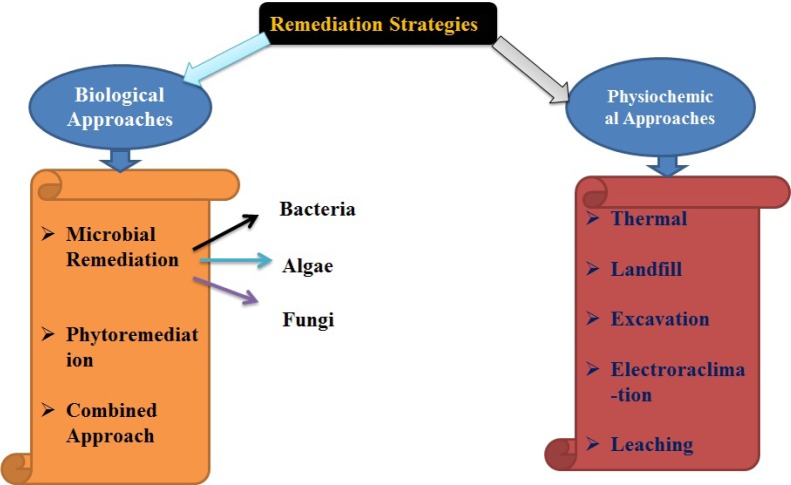
Classification of remediation strategies for heavy metals and other trace elements

To overcome such problems, using different mechanisms that plants must be able to keep ions hemostasis in their tissue by detoxifying the adverse effects of heavy metals. The different strategies for the remediation of heavy metals and other trace elements are shown in Figure 1(Fig. 1). The presence of organic products, like phytochelatin and metallothioneins inside the cell, having high affinity for absorption of heavy metals, can control their cellular concentration (Miransari, 2015[11]). Even phytoremediation, bioremediation and bioengineering will help to lessen the bioaccumulation of metals in the plants.

The future importance of bacterium UD1023 characterized as naturally occurring in the soil by Harsh Bais and Janine Sherier of the University of Delaware's, Department of Plant and Soil Sciences (Sohn, 2014[15]). UD1023 was identified for the first time in the soil of rice (*Oryza Sativa*) field in California by Bais (2006[3]). The basic function of UD1023 is to create an oily iron layer around the root of the rice that acts as a barrier for uptake of arsenic to grains. This bacterium is naturally present in the rhizosphere region of the soil that is enriched with soil microorganisms (Sohn, 2014[15]). Arsenic is found in the environment (soil, water) and also in food chain contaminants, specifically in rice that cause deleterious effects on human health in high rice consuming peoples (Zhao et al., 2010[20]). In addition some other heavy metals have also been reported recently to induce endocrine disorders in humans (Maqbool et al., 2015[10]). The preliminary study showed that UD1023 collect iron from the soil around the roots and slow down the arsenic uptake. The researchers have not yet determined that how exactly this process works and how much percentage of arsenic level is reduced through this process. Harsh Bais proposed hypothesis that roots drive out the oxygen, which oxidize the iron in the soil, making iron rust around the roots that prevent arsenic from binding and it outside of the plants. The complex and keeping ecological process occurs in the rhizospere region of the soil in which root exudates also help in signaling events, plant roots and other plants interaction (Storrs, 2014[16]; Bais et al., 2006[3]; Sandle, 2013[14]). Furthermore, Scientists have discovered the transporter proteins to help in carrying of heavy metals and metalloids into the roots. If they identify the gene responsible for the action of transporter proteins, then the flow of arsenic to the roots can be blocked. If researchers control or fix the arsenic uptake in the rice plants it might be helpful in the future because in the Chinese population 60 % arsenic come from rice (Sohn, 2014[15]). 

This work is under study and the researchers want to determine the mechanisms involved in the slowdown of arsenic movement into the roots and also to the edible portion of the plants by the UD1023. Our idea about this bacterium is related to scientists achievement in future, when they will successfully find the mechanism, how it mobilize iron and diminish the uptake of not only arsenic but also other noxious heavy metals. Other need of the hour is that scientists should successfully modify the roots of the rice and other plants as well as the soil with this bacterium. Then it will be helpful in practical application in agriculture. Coating seeds with this bacterium will have great importance if we utilize the low cost approaches and easy implementation to take a negligible level of arsenic in the human food chain. And we are of the opinion that this approach might be helpful in confining the uptake of other metals like cadmium, lead and mercury.

## Figures and Tables

**Table 1:**
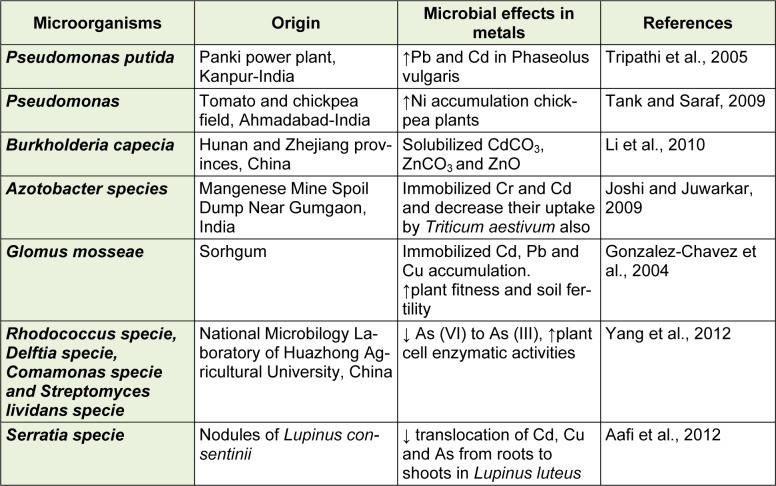
The role of soil microorganisms characterized for their potential role to reduce/immobilize the heavy metals and other trace elements
